# Nodular Sarcoid Myositis Treated With Hydroxychloroquine Monotherapy

**DOI:** 10.7759/cureus.41000

**Published:** 2023-06-26

**Authors:** Oksana V Mayovska, Elliot D Rosenstein, Neil Kramer

**Affiliations:** 1 Internal Medicine, Morristown Medical Center, Morristown, USA; 2 Rheumatology, Overlook Medical Center, Summit, USA; 3 Rheumatology, Atlantic Health System, Morristown, USA

**Keywords:** monotherapy, nodular myositis, myositis, sarcoidosis, hydroxychloroquine

## Abstract

Clinically significant granulomatous inflammation of skeletal muscle in sarcoidosis is rare. Glucocorticoids are generally considered the first-line treatment of sarcoidosis, but due to their side effect profile, the addition of steroid-sparing regimens has become increasingly more common. We report a patient with nodular sarcoid myositis who was successfully treated with antimalarial hydroxychloroquine alone. Whereas antimalarials have been reported to be an effective treatment of various organ involvement in sarcoidosis, to our knowledge, this is the first report of hydroxychloroquine monotherapy successfully treating nodular sarcoid myositis. Hydroxychloroquine monotherapy may be a reasonable initial treatment option for nodular sarcoid myositis and other forms of muscular sarcoidosis, as well as for other non-acute organ-threatening manifestations of the disease.

## Introduction

Sarcoidosis is a multisystem inflammatory disease of unknown etiology that is histologically characterized by the presence of non-caseating epithelioid granulomas. Granulomatous involvement occurs predominantly in the lungs but may involve any organ system, resulting in diverse presentations and a broad spectrum of disease severity. Histologic evidence of skeletal muscle involvement can be seen in 50-80% of patients with sarcoidosis, but only 0.5-2.3% of patients are symptomatic [1]. Three clinical subtypes of muscular sarcoidosis have been described: chronic myopathy, acute myositis, and nodular myositis [2]. Nodular sarcoid myositis (NSM), the rarest form of muscle involvement, presents with palpable nodules of various sizes, which may be tender or painful and can be associated with muscle weakness [1]. The treatment of all forms of sarcoid myopathy typically includes the use of glucocorticoids (GC) as first-line therapy with the use of “steroid-sparing” agents (methotrexate, azathioprine, leflunomide, tumor necrosis factor-α inhibitors) in patients exhibiting GC unresponsiveness or toxicity. The antimalarial drugs, chloroquine (CQ) and hydroxychloroquine (HCQ) have been used not only as steroid-sparing drugs but also as monotherapy in the treatment of specific end-organ involvement [3-13]. We report here a patient with NSM who demonstrated complete clinical and imaging resolution following treatment with HCQ alone.

## Case presentation

A 54-year-old woman from Taiwan had been well until age 37 when she was found to have a 2 cm mass in the right kidney during the evaluation of recurrent urinary tract infections. She denied fever or other constitutional symptoms. There was no history of respiratory, cutaneous, or ocular inflammation and no symptoms of sicca syndrome.

A preoperative computed tomographic scan of the chest revealed right hilar, subcarinal, and left para-aortic lymphadenopathy. Mediastinoscopy with lymph node biopsy revealed noncaseating granulomas, consistent with sarcoidosis. She underwent a radical right nephrectomy with findings of chromophobe renal cell carcinoma and had no cancer recurrence. Two years later, she developed progressive non-tender swelling of both calves with discomfort after prolonged standing. Magnetic resonance imaging (MRI) of the calves demonstrated multiple, bilateral areas of focal signal intensity involving the muscles of the superficial and deep posterior compartments and the anterior compartments. A fine-needle aspirate biopsy of the right gastrocnemius muscle confirmed the presence of noncaseating granulomatous inflammation. Special stains for microorganisms were negative. No therapy was instituted at that time.

She was re-evaluated after another 2 years complaining of progressive swelling and discomfort of the calves. Physical examination was notable only for an enlargement of the lacrimal and parotid glands bilaterally, nodular swelling, and the appearance of pseudohypertrophy of both calves.

**Figure 1:**
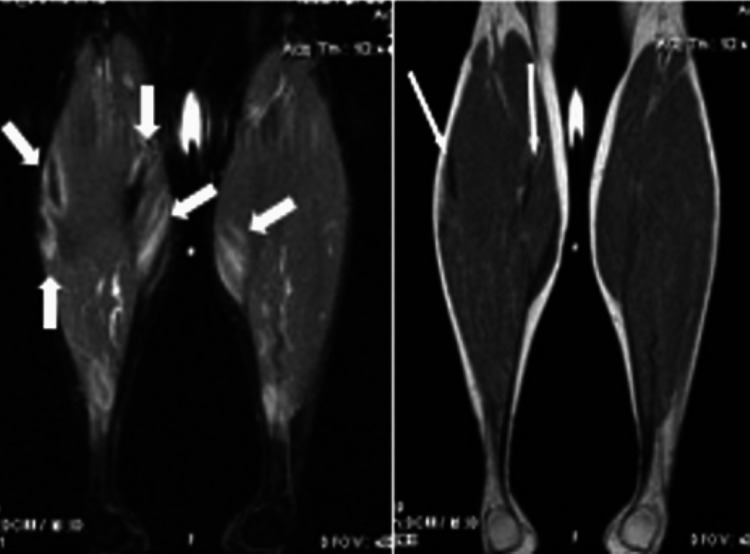
Magnetic resonance imaging (MRI) of both calves prior to therapy Coronal T2 short-tau inversion recovery (STIR) image of both calves, on the left, demonstrating increased signal within the gastrocnemius muscles bilaterally oriented along the muscle fibers. Some areas have central low signal. Corresponding coronal T1-weighted images, on the right, demonstrating only the central low signal areas.

Repeat MRI of the calves (Figure 1) showed areas of low signal intensity on T1-weighted images and central low signal intensity with surrounding high-intensity edema on inversion recovery images within the gastrocnemius muscles, consistent with NSM, which progressed since her prior study. Laboratory data were significant for angiotensin-converting enzyme levels of 95.2 U/L (normal: 8.0-52.0), creatine phosphokinase (CPK) 85 IU/L, and calcium 10.1 mg/dL. 

**Figure 2:**
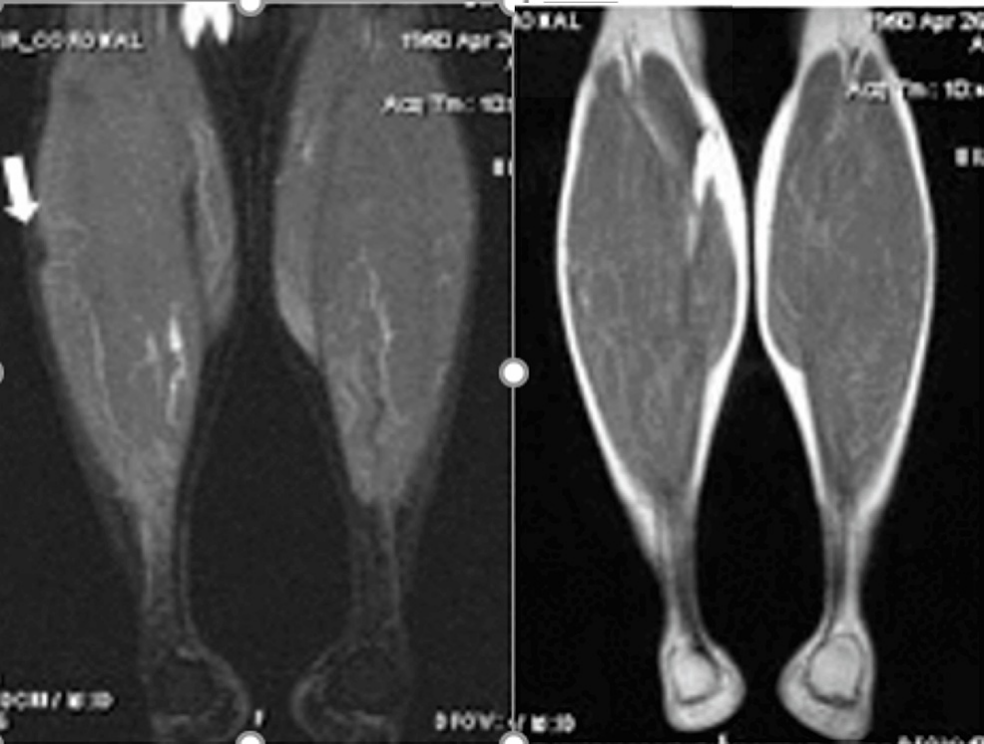
Magnetic resonance imaging (MRI) of both calves after 15 months of therapy Coronal T2 short-tau inversion recovery (STIR) image of both calves, on the left, demonstrating resolution of abnormal increased signal and subtle residual low signal. Corresponding coronal T1-weighted images, on the right, do not demonstrate any signal abnormality.

Due to the patient's fear of GC side effects a therapeutic trial of HCQ 6.5 mg/kg daily was started without concomitant GC. She experienced a gradual and complete improvement of her calf pain and swelling. A repeat MRI of the calves performed after 15 months of therapy (Figure 2) showed a resolution of NSM. The patient discontinued HCQ after 10 years of therapy and had no recurrence of calf nodules or other manifestations of sarcoidosis in the subsequent 3 years. 

## Discussion

NSM, the rarest subtype of musculoskeletal sarcoidosis, is characterized by the presence of multiple intra-muscular nodules, typically oriented longitudinally along muscle fibers. NSM manifests as palpable mass-like lesions within the muscle and can be accompanied by pain or tenderness, weakness, and sometimes with systemic symptoms such as fever, weight loss, and fatigue. The lesions predominantly affect the lower extremities and are commonly bilateral. NSM is seen more frequently in men and in younger individuals. NSM rarely remits spontaneously [1]. MRI findings are characteristic and demonstrate well-demarcated nodules with a central star-shaped area of hypointensity surrounded by areas of increased T1 and T2 signal in the periphery. The reduced MRI signal in the center of nodules may reflect fibrosis with loss of muscle fibers in areas that may be resolved by granulomas [14]. Histopathological examination reveals dense central fibrosis surrounded by granulomatous inflammatory changes with lymphocytes and epithelioid noncaseating granulomas and giant cells [2].

Antimalarials have been used since the early 1960s for the treatment of pulmonary [4,5], neurologic [7], cutaneous [8-10], and musculoskeletal manifestations of sarcoidosis, as well as for hypercalcemia of sarcoidosis [15]. The anti-inflammatory effects of these medications are believed to be due to the inhibition of toll-like receptor and stimulator of interferon gene (STING) signaling, resulting in reduced cytokine production; as well as inhibition of lysosomal activity preventing major histocompatibility complex (MHC)-class II autoantigen presentation [16]. Due to its better safety profile, especially in terms of potential retinal toxicity, HCQ has supplanted CQ for the treatment of inflammatory and autoimmune diseases. The recommended dose of HCQ is 200-400 mg daily (maximum 5 mg/kg daily) and that of CQ is 250 mg daily. Long-term use of HCQ, as is recommended for the treatment of systemic lupus erythematosus, has become the standard of care for that disease, with an excellent safety profile. Retinal toxicity after prolonged use is the most feared complication, with an overall frequency of 4.3%, occurring in 1% in the first 5 years, 1.8% after 6-10 years, 3.3% after 11-15 years, and 11.5% after 16-20 years of treatment. Cardiomyopathy and neuromyotoxicity are rare complications of HCQ therapy. HCQ can be used safely during pregnancy [16].

Current treatment guidelines for organ-threatening sarcoidosis recommend GC as first-line therapy, with the addition of immunosuppressive therapy (methotrexate, azathioprine, mycophenolate mofetil, infliximab) for GC-resistant cases or as steroid-sparing agents. Addition of antimalarials to GC is also recommended for cutaneous sarcoidosis [17]. Their efficacy when given as monotherapy has also been documented. CQ monotherapy, at a high dose of 250 mg twice daily in 43 patients with pulmonary sarcoidosis showed better and more rapid rates of radiographic improvement in comparison with 285 patients not receiving CQ. A high rate of relapse, usually occurring within 2 months of discontinuation, suggests that radiographic improvement did not simply represent the natural history of the disease [3]. Similarly, in a randomized, placebo-controlled trial of patients with symptomatic pulmonary sarcoidosis, 4 months of high-dose CQ therapy (600 mg for 8 weeks followed by 400 mg for 8 weeks) was associated with significant improvement in radiographic findings and a meaningful improvement in pulmonary symptoms compared to placebo, but the improvement did not persist at the 1-year mark [4]. In another randomized controlled trial, therapy with CQ, used as maintenance therapy for 6 months (750 mg daily, reduced by 250 mg every 2 months), reduced the rate of relapse and slowed the decline in pulmonary function [5]. In a retrospective analysis of 297 patients with sarcoidosis-associated uveitis, HCQ monotherapy (400 mg daily) achieved success in 11 of 29 patients [6]. HCQ, when used as monotherapy, has also been reported to show improvement in cutaneous sarcoidosis [8-10], as well as subcutaneous sarcoidosis [11], although cutaneous lesions usually returned upon discontinuation of therapy. Individual cases of neurosarcoidosis [7], paranasal sarcoidosis [12], and orbital myositis [13] responding to HCQ monotherapy have also been reported.

## Conclusions

To our knowledge, this is the first reported case of NSM successfully treated with HCQ monotherapy. Given the prolonged duration of NSM prior to initiation of therapy with HCQ, as well as 3 years of sustained resolution after HCQ discontinuation, we believe that remission of NSM was due to drug therapy rather than to spontaneous improvement. In view of the reported benefit of therapy with antimalarials for varied manifestations of sarcoidosis, it seems reasonable to consider monotherapy with one of these agents for NSM and potentially for other forms of sarcoid myositis, as well as for other non-acute organ-threatening manifestations prior to use of GC or other immunosuppressive therapies.
